# Using diffusion MRI to discriminate areas of cortical grey matter

**DOI:** 10.1016/j.neuroimage.2017.12.046

**Published:** 2018-11-15

**Authors:** Tharindu Ganepola, Zoltan Nagy, Aurobrata Ghosh, Theodore Papadopoulo, Daniel C. Alexander, Martin I. Sereno

**Affiliations:** a Department of Cognitive, Perceptual and Brain Sciences, UCL, London, UK; b Centre for Medical Image Computing, Department of Computer Science, UCL, London, UK; c Laboratory for Social and Neural Systems Research, UZH, Zurich, Switzerland; d Department of Psychology and Neuroimaging Centre, SDSU, San Diego, USA; e Project Team Athena, INRIA Sophia Antipolis—Meģditerraneģe, Nice, France

**Keywords:** Cortex, Cortical surface, Architectonics, Grey matter, Parcellation, HARDI, dMRI, Supervised leaning

## Abstract

Cortical area parcellation is a challenging problem that is often approached by combining structural imaging (e.g., quantitative T1, diffusion-based connectivity) with functional imaging (e.g., task activations, topological mapping, resting state correlations). Diffusion MRI (dMRI) has been widely adopted to analyse white matter microstructure, but scarcely used to distinguish grey matter regions because of the reduced anisotropy there. Nevertheless, differences in the texture of the cortical 'fabric' have long been mapped by histologists to distinguish cortical areas. Reliable area-specific contrast in the dMRI signal has previously been demonstrated in selected occipital and sensorimotor areas. We expand upon these findings by testing several diffusion-based feature sets in a series of classification tasks. Using Human Connectome Project (HCP) 3T datasets and a supervised learning approach, we demonstrate that diffusion MRI is sensitive to architectonic differences between a large number of different cortical areas defined in the HCP parcellation. By employing a surface-based cortical imaging pipeline, which defines diffusion features relative to local cortical surface orientation, we show that we can differentiate areas from their neighbours with higher accuracy than when using only fractional anisotropy or mean diffusivity. The results suggest that grey matter diffusion may provide a new, independent source of information for dividing up the cortex.

## Highlights

• Diffusion MRI provides architectonic contrast between a variety of cortical areas.
• dMRI provides complementary information in areas of low myelin content.
• High order rotational invariants provide more reliable parcellation results.

## Introduction

Early studies of the microstructure of the human cerebral cortex revealed a laminar structure comprising of six layers of varying thickness, and cellular and axonal fibre composition (Berlin, 1858, Lewis and Clarke, 1878, Mountcastle, 1997). The heterogeneous appearance of these layers, as well as differences in vertical and tangential fibre arrays in different parts of the cortical sheet, suggested that there might be a relationship between microstructural organisation and local functional specificity. Pioneers in this field (Brodmann, 1909, Campbell, 1905, Vogt and Vogt, 1919, von Economo and Koskinas, 1925) published hemisphere-wide maps demarcating the boundaries of cyto- and myeloarchitectonic domains based on sectioning and histological staining of cadaver brains. Those maps divided the cortical sheet into a complex mosaic based on radial and lateral variations in tissue composition. However, these somewhat incompatible parcellations were subject to many methodological criticisms: (1) their labour intensive nature limited sample size, which was problematic given inter-subject variability of cytoarchitectonic boundaries but also within-area variation (Geyer et al., 2000, Geyer et al., 1997, Orban et al., 2004, Sereno and Tootell, 2005, Wandell et al., 2007), (2) the unavoidable artefacts of the histological process, such as idiosyncratic plastic deformation and tearing of sections, (3) observer bias, and (4) a single tissue contrast per sample.

*In vivo* image-based methods for analysis of the grey matter have the potential to alleviate or eliminate some of these limitations. These methods are able to tackle inter-subject variability through the comparable ease of *in vivo* data collection. They can also be combined with additional multi-modal data from the same subject, to directly assess structure-function relationships, and lend themselves gracefully to observer-free algorithmic analyses. Some of these improvements have been applied to *ex vivo* data using observer independent intensity analysis (Amunts et al., 2000, Amunts et al., 1999, Bludau et al., 2014, Eickhoff et al., 2006, Geyer et al., 1996, Roland and Zilles, 1994, Schleicher et al., 2005, Zilles et al., 2002). However, despite their resolution advantages, such works are still labour intensive and lack the flexibility offered by a potential *in vivo* pipeline.

Thus far, image-based studies of the cortex have focused mainly on the analysis of myelin density via quantitative T1/R1 mapping (Dinse et al., 2015, Fischl et al., 2004, Geyer et al., 2011, Sigalovsky et al., 2006, Waehnert et al., 2016), R1 mapping in relation to map structure (Dick et al., 2012, Sereno et al., 2013), T2* mapping (Cohen-Adad, 2014, Sánchez-Panchuelo et al., 2012), MRT (Sánchez-Panchuelo et al., 2014) and the T1-weighted over T2-weighted ratio (Glasser et al., 2014, Glasser and Van Essen, 2011). However, these proxies for myelin density provide only a single-dimensional description of variation in cortical microstructure. Hence, using T1 as a sole marker is less informative in areas with low, relatively uniform myelination that are found outside primary and secondary sensory and motor cortices (Ganepola et al., 2017, Glasser et al., 2014). A recent multidimensional approach has added resting state and task-based fMRI data to the T1w/T2w myelin proxy to generate a full-hemisphere cortical parcellation (Glasser et al., 2016). This method does not attempt to directly measure the fine-grained structural characteristics of cortical cytoarchitecture, instead relying more heavily on functional information for much of the cortex. It therefore is not exactly analogous to the traditional parcellation maps discussed above, e.g., Brodmann (1909), and may be less suited for assessing structural changes underlying abnormal brain function in higher-level areas.

Diffusion magnetic resonance imaging (dMRI) has become ubiquitous in the study of white matter (WM) microstructure (Basser et al., 1994, Le Bihan, 2003, Le Bihan et al., 2001). By measuring the displacement of water molecules within tissue compartments, dMRI offers *in vivo* insight into structural properties of microenvironments, such as WM fibre orientation (Douek et al., 1991), fibre fanning and dispersion (Sotiropoulos et al., 2012, Zhang et al., 2012, Zhang et al., 2011), the volume fractions of various tissue types (Jeurissen et al., 2014), and axon diameter (Alexander et al., 2010, Assaf et al., 2008, Zhang et al., 2011). Although dMRI and T1 are both affected by similar structures (e.g., myelinated axons), dMRI can provide a multi-dimensional feature space that has increased potential to distinguish differences in local architecture. Two different cortical regions or layers might contain the same total amount of myelin, but that myelin might be arrayed differently. For example, one area might have more radial than tangential fibres, or these areas may have different tortuosity.

Initial grey matter (GM) applications of dMRI focused on the developing brain due to its increased anisotropy (Gupta et al., 2005, McKinstry et al., 2002, Mukherjee et al., 2002). Others have used tractography to subdivide the cortex based on the WM connectivity between regions (Anwander et al., 2007, Behrens and Johansen-Berg, 2005, Jbabdi et al., 2009, Johansen-Berg et al., 2004, Moreno-Dominguez et al., 2014, Ruschel et al., 2014). Compellingly, several papers have demonstrated a good correspondence between cortical histology and dMRI using *ex vivo* data (Bastiani et al., 2016, Leuze et al., 2014, McNab et al., 2009). For example, Aggarwal et al. (2015) used spherical deconvolution at 90 μm^3^ resolution to show layer specific changes in diffusion orientation between different functional areas, including area-defining features such as the Stria of Gennari in the primary visual cortex (V1).

Imaging of the microscopic details that define cortical areas at *in vivo* resolutions has recently become plausible through advancements such as simultaneous multi-slice acquisition, improved gradient systems, better motion/eddy current correction algorithms, and ultra-high field MRI (Heidemann et al., 2012, Heidemann et al., 2010). Some have combined diffusion tensor imaging with cortical surface-based analysis to successfully demonstrate differences between the primary motor (M1) and somatosensory (S1) cortices (Anwander et al., 2010, McNab et al., 2013). Others have extended these findings by applying similar features to the medial surface of the cortex, with the aim of understanding how the microstructure of the cortex adapts when it folds (Kleinnijenhuis et al., 2015). Calamante et al. (2017) estimated the apparent fibre density across the cortical sheet, reporting region specific changes that correlate with known patterns of myeloarchitecture. These works demonstrate that HARDI techniques can effectively capture microstructural changes between cortical regions, potentially making *in vivo* cortical parcellation possible. Crucially they all relied on surface-based analysis to circumvent the difficulties of cortical folding, and in some cases, to provide laminar-like analysis. Yet very few attempts have been made to characterise the small but detailed changes in signal expected to result from the different architectonic tissue textures found in different cortical areas (Ganepola et al., 2017, Haroon et al., 2010, Nagy et al., 2013). Neither have there been any attempts, so far, to compare any of the multitudinous WM dMRI techniques.

In this paper, we investigated the extent to which HARDI data can be used to discriminate cortical areas. We performed a set of classification tasks, comparing the efficacy of several different diffusion-based feature sets. These included popular WM methods, e.g., the diffusion tensor, as well as higher-order, non-parametric approaches such as spherical harmonic invariants. In addition, we determined which cortical areas could be reliably distinguished from their neighbours by developing a supervised learning classification framework that utilized the 180 cortical areas defined by Glasser et al. (2016) as prospective training labels. We objectively quantified regional differences across the whole cortical surface, whereas previous works have focused on a smaller selection of areas. We present results at both the individual and group-level and demonstrate that regionally specific contrast is present in diffusion datasets for the majority of the cortex.

## Methods

### Data and pre-processing

Data sets for 40 subjects were obtained from Human Connectome Project (HCP) Q3 500-Subjects release, specifically, subjects were selected from the unrelated participants subset. Data were from healthy participants and publicly available under ethics approval. In-depth descriptions of the acquisition parameters and pre-processing pipelines are provided in the HCP documentation (Glasser et al., 2013, Sotiropoulos et al., 2013, Ugurbil et al., 2013, Van Essen et al., 2013). In summary, data were collected on a custom Siemens 3T Skyra system (*G*_max_ = *100 mT/m*). Diffusion datasets had 270 gradient directions across three b-shells, *b*=*1000, 2000 and 3000 s/mm*^*2*^, with twelve *b*=*0* s/mm^2^ images interspersed. A spatial resolution of *1.25 mm*^*3*^ was achieved using multiband accelerated imaging. Pre-processing steps conducted prior to data release included eddy current and motion correction (Sotiropoulos et al., 2013).

### Surface reconstruction and sampling

We utilized the FreeSurfer Pipeline HCP script to produce surface meshes for each subject. This improved pipeline was chosen over the standard recon-all pipeline to make use of the high-resolution (*0.7 mm*^*3*^) T1w and T2w structural scans which help reduce surface placement errors (Glasser et al., 2013).

The HARDI data of each subject were sampled onto their cortical surface reconstruction using the procedure from Nagy et al. (2013). The average b0 image was registered to the T1w volume using an affine transformation matrix. The same transformation matrix was then applied to the DWIs. The signal intensity for each DWI image was nearest-neighbour sampled at the midpoint between the GM/WM boundary surface and the pial surface (i.e., cortical depth = 0.5). Equidistant sampling was used to minimise partial volume contamination from either the subarachnoid space or the white matter. We tested an approximation of the more anatomically realistic equivolume sampling (Bok, 1929, Waehnert et al., 2014), but as a consequence of the relatively low spatial resolution, nearest-neighbour sampling and single depth analysis, we observed little difference between the two approaches.

### Feature sets

Four different diffusion MRI analysis techniques were used to generate six feature sets (see Table 1) that were tested in the classification experiments below. We selected the feature sets to address whether either of the following is beneficial when differentiating cortical areas: (a) projecting the HARDI signal characteristics into the local frame of reference, and (b) increasing the model complexity. The four techniques are described below.

1. The diffusion tensor (DT) (Basser et al., 1994, Basser and Pierpaoli, 1996) provides two scalar metrics, mean diffusivity (MD) and fractional anisotropy (FA), as well as directional information stored in its three eigenvectors. One simple way to generate surface-specific information from the DT is to measure the dot product of the primary eigenvector and the local surface normal (i.e., the radiality index, RI (McNab et al., 2013)). This is a scalar metric that indicates the extent to which diffusion is radial (e.g., along apical dendrites of pyramidal cells).
2. The neurite orientation, dispersion and density imaging (NODDI) (Zhang et al., 2012) is a popular WM model which aims to increase specificity to microstructural properties. It disentangles FA into the possibly more anatomically relevant neurite density index (NDI), and orientation dispersion index (ODI). In addition, NODDI provides the isotropic volume fraction (V_iso_), representing free-water content.
3. In the third approach, we more finely characterised the shape of the local diffusion surface by first decomposing the HARDI data into a 6th order spherical harmonic (SH) series. A total of 9 features were generated for each b-shell. The first 4 features are fully rotationally invariant, whereas the last 5 are invariant in the local tangent plane to the cortical surface. Features 1–4 were the k = 1,2,3,4th moments of the apparent diffusion coefficients (ADCs). The 5th feature was the mean of the ADC in the direction of the surface normal, and the remaining 4 features were the k = 1,2,3,4th moments of the ADC in the plane that is parallel to the cortical surface. A related approach has already been tested on cortical tissue (Ganepola et al., 2017, Nagy et al., 2013).
4. A final approach used a 4th order tensor representation of the ADC and all its informatically (or functionally) complete and irreducible invariants. These invariants fully describe the geometric characteristics of the ADC up to any orientation or pose in 3D. Higher order tensors were introduced as an alternative (and bijective) mathematical basis to spherical harmonics in Özarslan and Mareci (2003). The tensors were estimated using the ternary quartic (TQ) framework to ensure positive ADC as in (Barmpoutis et al., 2009, Ghosh et al., 2014), while the invariants were computed following the method proposed in Papadopoulo et al. (2014). The invariants were found by progressively projecting the TQ coefficients via an orthogonal transform and a rotation transform to a canonical representation with 12 degrees of freedom.

**Table 1:** The names and descriptions of each of the feature sets utilized in the classification experiments. The number at the end of each name signifies the total dimensionality (length) of each feature set.

Feature Sets	Description
DT3	[MD, FA RI] calculated after fitting the diffusion tensor to the b = 1000 s/mm^2^ data.
DT9	[MD, FA RI] ×3 After fitting DT to each b-shell separately and concatenating the 3 metrics from each shell.
DT6	[MD, FA] ×3 Same as DT9 with the radiality index omitted i.e. no surface normal component.
ND3	[NDI, ODI, V_iso_] After Fitting the NODDI model to the full multi-shell dataset.
SH27	9 features per b-shell calculated from the SH series. First four features are fully rotationally invariant, the remaining five are invariant in the plane perpendicular to the local cortical surface normal.
4T36	12 features per b-shell, calculated from the 4-tensor, creating a functionally complete set of rotational invariants.

The latter two approaches better characterise the precise shape of the HARDI signal due to their sensitivity to higher-order details. Because GM regions have much lower anisotropy than WM, which often consists of coherently organised WM fascicles, we hypothesised that these higher order features might be better suited to capturing the complex and subtle variations that are known to distinguish different cortical areas.

### Classification experiments

#### Binary classification between S1 and M1

The S1 and M1 areas are very distinct from each other and consistently located across subjects. Therefore, classification between these two regions was chosen as a robust initial test-bed. Training labels were defined for each subject using the HCP multi-modal parcellation (HCP-MMP) atlas (Glasser et al., 2016). Specifically, the labels 3b, and 4 were registered from the fsaverage surface to each subject's surface tessellation (Fischl et al., 1999).

Random forest classification (RFC) (Breiman, 2001) was implemented in sk-learn (*forest size*=*20*, *tree depth*=*7*, other parameters at default values) to distinguish the two areas using a pool of 20 subjects. Data under the two regions were extracted for each feature set in each subject. The classifier was trained on a set of subjects for several training group sizes (TS), ranging from 1 to 19 subjects, and then tested on an unseen subject. The same training group was maintained until all feature sets were tested. A leave-one-out approach was implemented to ensure that all of the available subjects were tested in turn for each TS.

We hypothesised that the low-order features would dominate the classification because they have previously been shown to be very distinct between S1 and M1 (Anwander et al., 2010, McNab et al., 2013).

#### Group average whole hemisphere parcellation

A hemisphere-wide parcellation pipeline was developed to test the DT6, DT9 and SH27 feature sets across a broader range of cortical areas. These three feature sets were prioritised because they encompass the differences we intended to test, i.e., DT9 includes surface specific features where DT6 does not, and SH27 is a higher-order method compared to DT9 and DT6. We also employed population averaging for these classification tests to increase the contrast-to-noise ratio between cortical areas.

Of the 40 HCP subjects, 30 subjects were selected as the training pool and the remaining 10 were assigned as the test pool. 6 of the 30 training subjects were randomly selected at a time, to generate a total of 20 group average training sets. Surface-based averaging (Fischl et al., 1999) was performed on each dimension (column) of the feature sets (Ganepola et al., 2017). The same process was repeated to create a test dataset from the test pool, which also averaged data from 6 subjects.

**Fig. 1:**
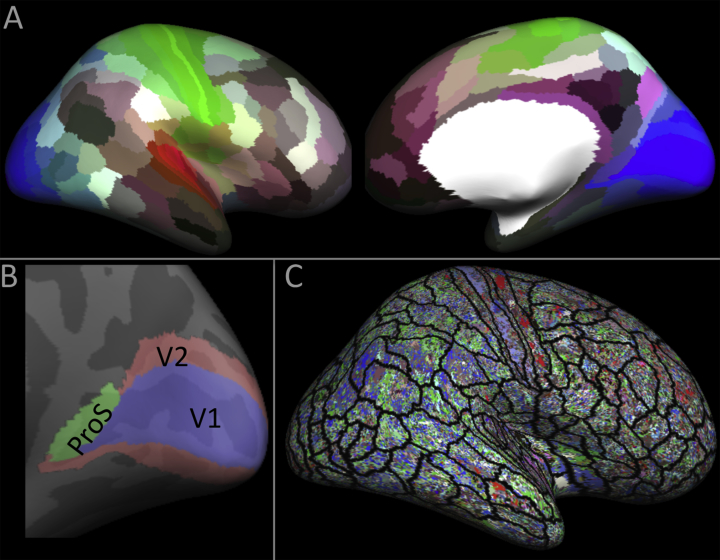
(A) The classification training labels from the Human Connectome Project multi-modal parcellation (Glasser et al., 2016). (B) An example of a neighbourhood of areas, in this case, for the classification of V1. (C) An example of a classification result where instead of the neighbourhood approach, a 180 area multiclass classification is attempted. The result was generated using the DT9 feature set, and shows very little structure.

The 180 areas of the HCP-MMP were utilized as a set of prospective hemisphere-wide, training labels. Given a test average, the cortical area marked by each of the training labels was tested in turn, using a multi-label RFC, against its neighbouring cortical areas. For example, when trying to predict the class of the data marked by the V1 label, the classifier was trained on data taken from 3 labels in the training averages: ProS (prostriata), V1 and V2 (Fig. 1B). This neighbourhood approach mimicked traditional parcellation techniques that define areas based on local transitions in laminar appearance, and also reduced the number of classes within a single test to a relevant set. Fig. 1C demonstrates the ineffectiveness of implementing a global 180-label classification experiment.

#### Single subject whole hemisphere parcellation

The methods presented above were combined to produce a whole hemisphere parcellation on an individual subject to assess whether between-area contrasts can be detected without averaging.

The neighbourhood multi-label RFC approach from the group average pipeline was used to classify the data marked by each of the 180 HCP-MMP areas for a single unseen subject. The training data was generated by concatenating the data for each of the neighbourhood labels from a group of training subjects, as in the binary M1/S1 tests. The training group was reduced to 10 subjects to reduce runtime and memory requirements of the classifier. During registration of the training labels, a small number of vertices (1–5%) were assigned to multiple classes. In each case, the vertex was assigned to its mode class label; in the absence of a mode class, the vertex assignment was selected randomly from its predicted classes.

### Searchlight cluster count

**Fig. A2:**
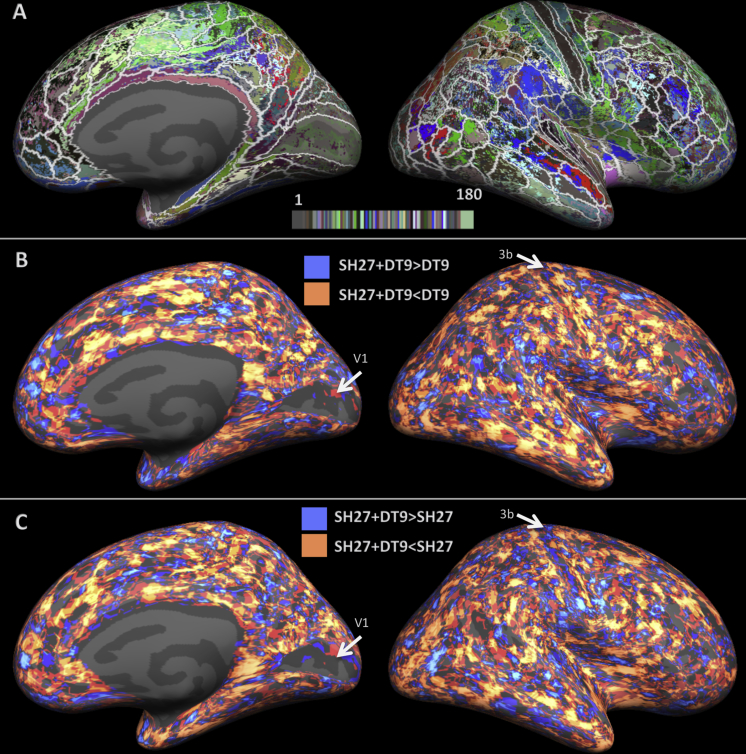
Group average classification results for the combination of SH27 and DT9 feature sets. (A) The parcellation result with the same colour assignment as Fig. 3B. Again, the HCP training labels have been overlaid in white. (B) The searchlight cluster coherence comparison between the combined feature set of SH27 and DT9 together vs DT9 alone. (C) The same as B but with comparison to the SH27 feature set alone. In both B and C, a lower cluster count indicates a more spatially coherent parcellation, therefore orange (lower value in SH27 + DT9) indicates that the combined feature set performed better.

A quantitative vertex-wise comparison method was developed to evaluate the quality of different full hemisphere parcellation results. Here, quality was defined as the local spatial coherence of the parcellation. Given two corresponding parcellation results, A and B, the number of unique cluster IDs within a 90-nearest neighbour surface searchlight surrounding each vertex were counted, and the resulting cluster counts were subtracted from each other (A - B). A positive value (A < B, orange in Fig. 4, Fig. 6, A2) denotes that A did better (i.e., local regions had fewer different cluster IDs) while a negative value (A > B, blue) indicates that B did better. The searchlight diameter was chosen to be somewhat smaller than the width of a typical cortical area.

## Results

### Binary classification of S1 vs M1

**Fig. 2:**
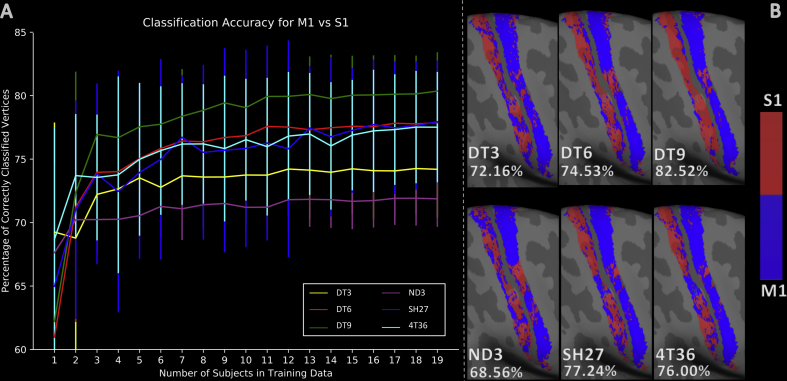
(A) The mean classification accuracy for each feature set, at each training group size (TS). Error bars are the standard deviation in classification accuracy across repeats for each TS. (B) M1 vs S1 classification results for a typical subject from the leave-one-out, TS = 19 test. Accuracy scores given as the percentage of correctly classified vertices. Red corresponds to the S1 class label and blue to the M1 class label.

The results for binary classification between S1 and M1 using different training group sizes are shown in Fig. 2A. The feature sets DT6, DT9 (incl. surface-based features), SH27 (incl. surface-based features) and 4T36 demonstrate a similar trend with steep improvement in accuracy from TS = 1 to TS = 3 followed by more gradual, improvement up to TS = 19. The DT3 and ND3 feature sets exhibit a more modest rate of improvement in accuracy between TS = 1 and TS = 3, and significantly lower plateaus. It is evident that using fewer than 3 training subjects does not provide a broad enough set of training examples to account for inter-subject variability; incorporating at least 10 subjects is beneficial.

The DT9 feature set performs best in the classification between M1 and S1 for all values of TS larger than 2, achieving a mean classification accuracy of **80.36%** at TS = 19. SH27, 4T36, DT6, DT3, and ND3 gave mean accuracies of **77.94%**, **77.51%**, **77.87%**, **73.98%**, and **71.87%** respectively, for the same TS. A Wilcoxon signed rank test between each set of results found the differences between DT9 and all other feature sets to be significant (p < .02), whereas the performance of SH27, 4T36 and DT6 were not significantly different from each other. ND3 and DT3 were also found to be significantly different from each other and all of the other feature sets (p < .001). Aside from the reasons given in the methods, we chose not to include DT3 and ND3 in subsequent experiments because they performed comparatively poorly here. 4T36 was also omitted because it performed so similarly to SH27.

In Fig. 2B it is evident that DT9 provides the most spatially coherent result, particularly within area S1.

### Group average whole hemisphere classification

#### Qualitative assessment

**Fig. 3:**
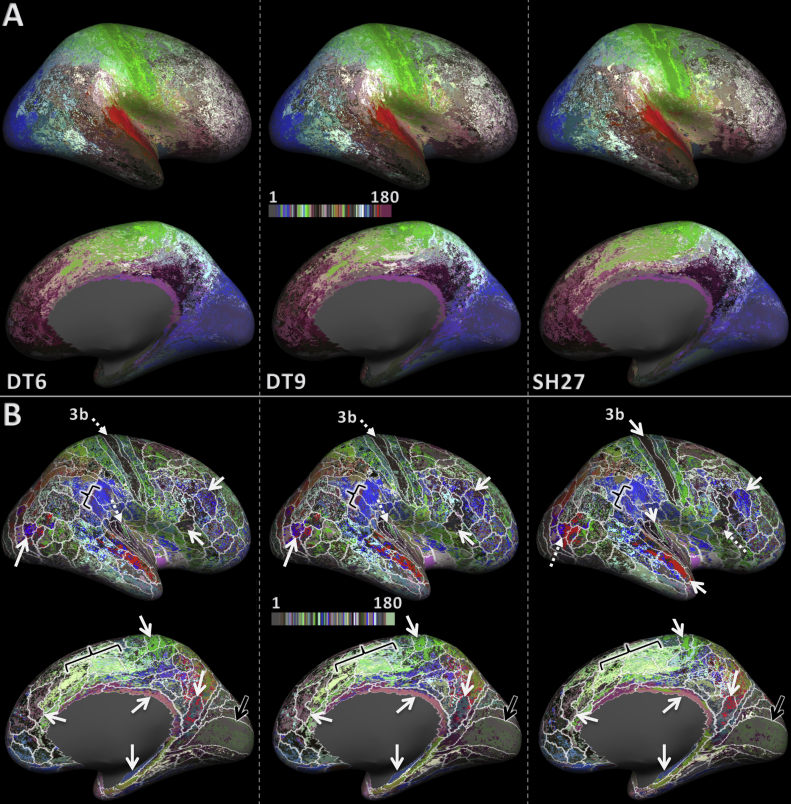
Maps of the group average whole hemisphere parcellation result for feature sets DT6, DT9, and SH27 (left to right). (A) Shows the original colour scheme from the HCP-MMP. (B) Shows the same results as A but with the colour scheme shuffled to achieve better contrast between neighbouring areas. In addition, the boundaries of the training areas are overlayed in white. The solid white arrows signify areas that have a large overlap with the training labels. The dotted white arrows indicate that an area is subdivided or not as well classified as it was for another feature set. The black arrows point to the V1 area that did not classify as well, despite its distinct architecture. The black brackets point out regions in which one cluster expands over several training labels.

The lateral and medial views of the parcellation result for the DT6, DT9, and SH27 feature sets are displayed in Fig. 3. In general, early sensory and motor areas showed a strong resemblance to the training labels, exhibiting spatially locally coherent clusters. Moving away from those easy-to-distinguish areas, the spatial coherence of the classification results was reduced, and a number of training areas contained a speckling of multiple cluster IDs. One can also observe several coherent clusters that contain several training regions (black brackets).

The overall trend comparing DT6, DT9, and SH27 (left to right) is an observable decrease in the granularity of the parcellation in some areas (e.g., area 3b). This suggests that inclusion of a surface normal component (DT9 & SH27 compared to DT6) and the use of higher order features (SH27 compared to DT6 & DT9) both provide useful additional information to the training and classification process.

Qualitative assessment of the lateral surface indicates that area 3b (part of S1) has the most distinct HARDI signal profile compared to its neighbouring areas, as all of the feature sets achieve a reasonable correspondence to the training label for this area. Some regions show markedly different classification outcomes between the different feature sets (dotted arrows). For example, the MT region is subdivided by SH27, but recovered more completely by DT6 and DT9. The SH27 subdivision may reflect inter-subject variability in the location of MT proper (Bridge et al., 2014).

Inspection of the medial surface surprisingly reveals that none of the feature vectors strongly differentiated the primary (V1) and secondary (V2) visual areas (black arrows). Areas close to the medial surface interface with the corpus callosum, e.g., retrosplenial complex and hippocampus, can be accurately classified by all feature sets and are known to be architectonically distinct from most other medial and lateral cortical areas.

#### Quantitative assessment

**Fig. 4:**
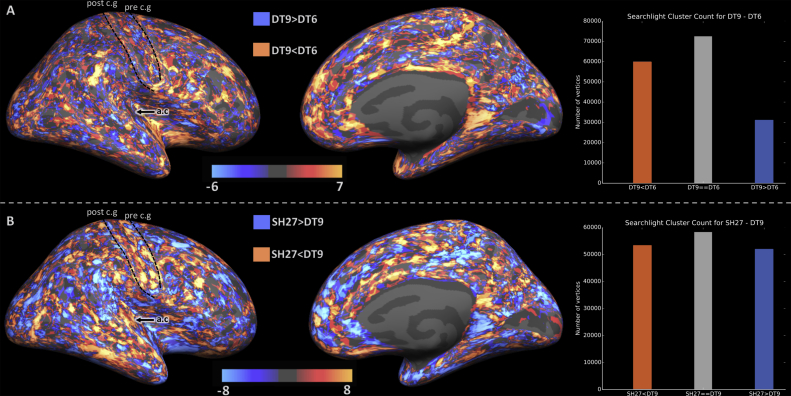
Searchlight cluster coherence results. (A) DT9 vs DT6: orange indicates that the parcellation was more spatially coherent in DT9 and blue indicates the reverse effect. (B) SH27 vs DT9: orange indicates that the parcellation was more spatially coherent in SH27 and blue indicates the reverse. Bar charts to the right show the number of vertices satifying each condition across the whole hemisphere. The dotted contours highlight the position of the pre-central gyrus (pre c.g) and post-central gyrus (post c.g), and the black arrow points to the auditory core (a.c).

The searchlight comparison of the results is shown in Fig. 4. Overall, DT6 and DT9 are similar to each other in terms of cluster coherence, as a large number of vertices (over 70000) had equal cluster counts for both feature sets. Where they differ, DT9 tends to out-perform DT6, with 60000 wins for DT9 (orange) compared to 30000. The local cluster counts of DT9 and SH27 are equal for a smaller portion of the cortex (just under 60,000 vertices). The number of vertices in which SH27 outperforms (orange) or underperforms (blue) DT9 are relatively even. However, the distribution of these results indicate that SH27 provides more spatially coherent clusters in the central sulcus, auditory core, MT, cingulate sulcus and the temporal lobe. In contrast, DT9 performs better in the inferior parietal lobe and posterior default mode network areas.

The bar plots in Fig. A1 of the supplementary material display the classification accuracy of each feature set in each of the 180 areas. 125 of the 180 areas were reproduced with a greater than chance accuracy for all three feature sets. Many areas were highly reproducible, for example, the Hippocampus, area 33pr and MT to name a few. Areas such as LIPv and MIP could not be distinguished from their neighbours.

**Fig. 5:**
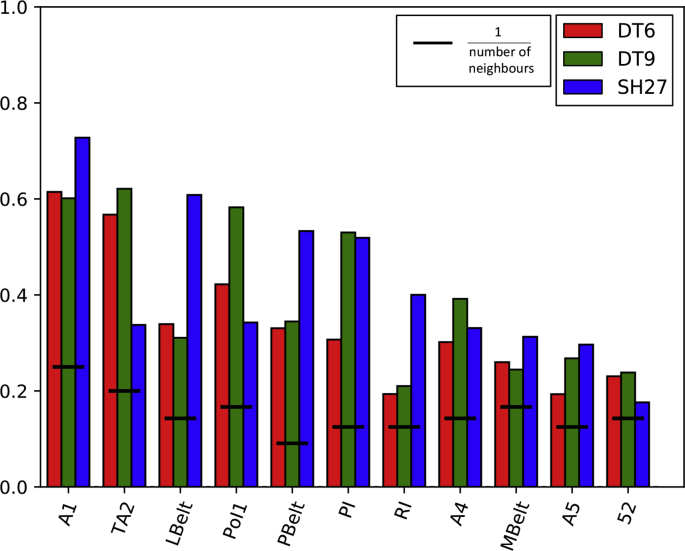
Bar graph comparing the classification performance in auditory areas. The bar heights indicate the porportion of correctly classified vertices in each ROI for the DT6 (red), DT9 (green) and SH27 (blue) feature sets. The black lines indicate the chance outcome for each ROI, i.e. 1/the number of neighbours for each ROI.

Fig. 5 takes a closer look at performance for a subgroup of areas belonging to the auditory network in the insular cortex. Many of the areas in the auditory subgroup are classified with a much higher than chance accuracy. SH27 is the winning feature set for just over half of the areas, whereas DT9 wins in the remaining portion. The performance of SH27 is better within the auditory core (A1, RI) and surrounding belt areas (LBelt, PBelt, MBelt), which have previously been shown to have extremely distinct myelin characteristics (Sereno et al., 2013). In contrast, DT9 yields higher accuracy in areas that are generally more architectonically uniform (areas outside of primary, secondary, and tertiary visual, auditory, somatosensory, and motor areas).

### Single subject whole hemisphere classifier

**Fig. 6:**
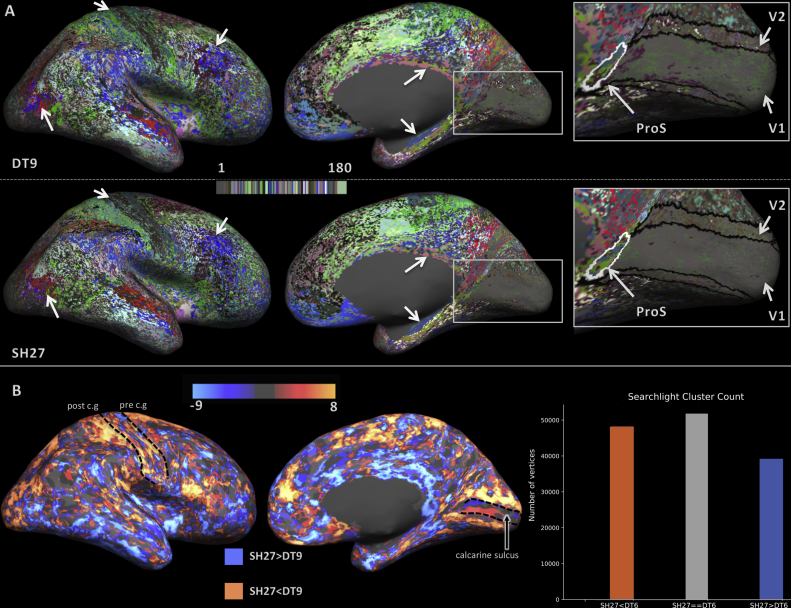
The single subject full hemisphere parcellation results. (A) Medial and lateral views of the parcellation for DT9 (top) and SH27 (bottom). The white arrows highlight areas that exhibit a good correspondence to the training labels. The right panel provides a close up view of the primary visual cortex. (B) Maps comparing searchlight cluster coherency between the single subject DT9 and SH27 parcellations. Orange indicates that the parcellation was more spatially coherent in SH27 and blue indicates the reverse. Bars on the right show the number of vertices belonging to each condition across the whole hemisphere. The dotted contours mark out the pre-central gyrus (pre c.g.), post-central gyrus (post c.g.) and calcarine sulcus.

Fig. 6 shows single subject whole hemisphere classification results for DT9 and SH27. The results for DT6 are not shown but the trends were similar to the group average result with DT6 giving the most granular, least accurate classifications. The overall spatial coherence is lower for both feature sets than the group average results (see Fig. 3). However, area-like clusters can still be observed in both (white arrows). The map for SH27 is qualitatively smoother than that of DT9. In particular, SH27 provides a much more coherent definition of V1 than does DT9, or indeed any of the group average results above. But again, neither of the feature sets manages to properly differentiate V1 from V2.

The quality of each parcellation was compared more rigorously using the searchlight cluster counting method (Fig. 6B). Again, as in the group average findings, SH27 provides a quantitatively smoother parcellation overall. The distribution of these results is also similar to the group average result, i.e., SH27 is more consistent in regions such as the sensorimotor areas of the central sulcus and the primary visual areas.

## Discussion

### Areas of high reproducibility

This work provides evidence that diffusion MRI is a sensitive and anatomically meaningful contrast for identifying differences between cortical areas at 3T resolutions. We demonstrated that the M1 and S1 cortices can be reliably distinguished from each other using a simple white matter model, the diffusion tensor, as previously suggested (McNab et al., 2013). The transition between S1 and M1, within the central sulcus, is one of the most distinct in the cortical sheet (Brodmann, 1909, Geyer et al., 1997, White et al., 1997). Furthermore, the associated Brodmann Areas, 3b (part of S1) and 4 (M1), are consistently located along the posterior and anterior banks of the central sulcus respectively. Thus, it is understandable that these areas were easy to reliably distinguish. However, accurate classification outcomes were not limited to these two regions. We found that 3b and several other areas can be reliably differentiated from their surrounding cortical tissue using group average data. For example, A1 and auditory belt areas demonstrated a large overlap with their corresponding training labels. High reproducibility in these areas suggests the ability of the classification method and feature sets to overcome the confounding effects of inter-subject variations in idiosyncratic cortical folding patterns within Heschl's gyrus (Leonard et al., 1998).

Areas of high reproducibility are not limited to myelin rich areas; for example, 78% of the vertices in the inferior frontal sulcus area, IFJa, were correctly assigned using the SH27 feature set despite this area having six neighbours. Such examples indicate that dMRI provides useful contrast in regions where myelin density is not as informative. However, as the fidelity of the training labels is questionable in these areas, further investigation will be required to illuminate what is driving classification outcomes in these regions. Nevertheless, the results suggests that dMRI could be a useful modality to incorporate in future studies that aim to non-invasively fingerprint the differing microstructure in cortical units. This hypothesis was further supported by the analysis of the single subject, whole hemisphere parcellation in which area-like clusters were demonstrated in similar regions to the group average results.

### Areas of low reproducibility

**Fig. 7:**
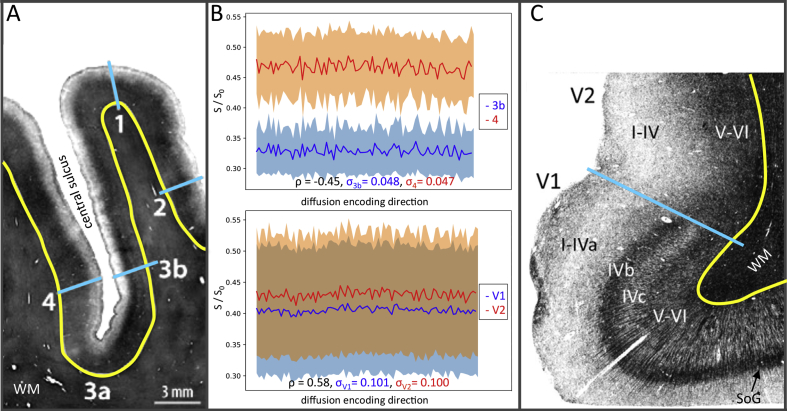
Myelin stains and DWI signal intensities from a selection of areas. (A) Myelin section from the central sulcus region, adapted from Dinse et al. (2015). (B) The mean DWI signal intensity in areas 3b and 4 (top) and V1 and V2 (bottom) for a single subject. The subject is the same as the one for which results were shown in Fig. 6. The signal intensities have been normalised by the mean b = 0 s/mm^3^ signal and the shaded regions indicate the standard deviation within each ROI. σ_area_ is the mean std across the DWIs. ρ, is the Pearson correlation coeffecient between the mean signals for each pair of areas. (C) Myelin section (Amunts and Zilles, 2015) depicting the boundary between the V1 and V2 regions. The blue lines in A and C mark the transitions between different cortical areas and the yellow contours mark the GM/WM boundary.

All feature sets failed to clearly distinguish the V1 and V2 areas in the group average classifier, despite the marked differences between these areas (Amunts et al., 2000, Hinds et al., 2009, Mountcastle, 1997). It is possible that inter-subject variability regarding the exact boundary between these two regions causes mixing of data when the averaging is performed which in turn obscures the contrast between these regions in both the training and test data. Although the horizontal meridian of V1 always resides within the calcarine sulcus, V1's extension onto the surrounding gyrus, and therefore its boundary with V2, shows considerable variation across subjects (Amunts et al., 2000). It is also likely that the relatively low resolution of the HCP data is insufficient to delineate defining characteristics in the extremely thin V1 region. Turner et al. (2008) have suggested that an isotropic resolution below 0.6 mm^3^ is required at 3T to consistently image Stria of Gennari (Fig. 7C). It may also be that the interacting effects of orientation dispersion and microstructural composition (Kaden et al., 2016, Reisert et al., 2017) diminishes differences in the dMRI signal between these two regions. Fig. 7B indicates that the signal intensity across different gradient directions is more correlated between V1 and V2 compared to S1 and M1.

In some regions, multiple training areas were classified as the same cluster (black arrows Fig. 3). It is possible that the dMRI signal is not sensitive to subtle differences between these regions or that the multi-modal training labels do not correspond to their architectonic subdivisions.

### Cluster coherence

**Fig. 8:**
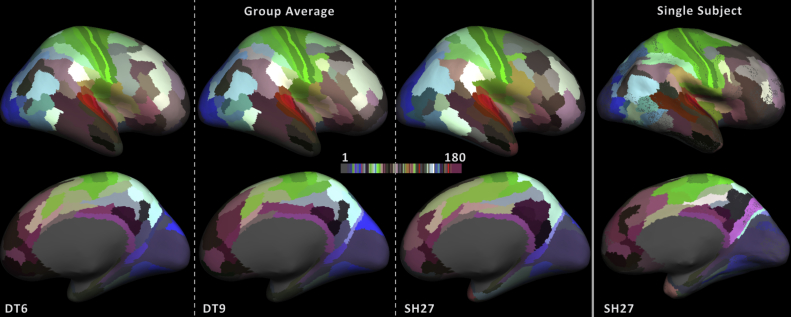
The winner takes all group average parcellation results for DT6, DT9 and SH27 and the single subject winner takes all results for SH27. For each feature set, the results were generated by calculating the most frequent class under each training label and assigning the entire region to that class ID. The resulting labels match the training labels for 106, 113, 105, and 99 (from left to right) out of the 180 areas.

It should be noted that no smoothing steps were implemented to enforce spatial coherence in any of the cortical maps illustrated so far. Of course, a much cleaner result can be obtained by adding additional post-processing steps. For example, a winner-take-all approach (Fig. 8) results in a significantly less noisy parcellation and closer correspondence to the training atlas. Though this may be beneficial in some applications, we felt that it was critical to illustrate the unaltered, vertex-wise results that will eventually form the basis for more complex, knowledge-based pipelines. Furthermore, the unsmoothed results allowed us to use cluster coherence as a measure of performance (Fig. 4, Fig. 6B). Importantly, this analysis suggested that the higher-order SH27 method provides a more spatially coherent result in areas where the training labels were defined using architectonic information, such as heavily myelinated primary cortices.

### Training labels

The HCP-MMP labels were generated from the simultaneous analysis of myelin density, resting state fMRI, and task fMRI. As such, the boundaries found by combining these modalities are not necessarily correlated with the cortical features that are captured by the dMRI signal attenuation. These provisional labels cannot be expected to exactly correspond to the underlying cortical areas, as defined by their architectonic properties alone. In addition, the uniform parcels of our training labels are themselves an abstraction from the real neocortex. For example, many of the best-defined cortical areas (e.g., MT, S1, M1) contain internal architectonic boundaries that are just as striking as any between-area boundary (see Kuehn et al. (2017) on M1/S1; Sereno et al. (2015) on MT). This is particularly important to consider when interpreting the results, especially given the supervised nature of the classification methods. Care should be taken not to over interpret the above results in regions where the training labels were heavily influenced by functional MRI modalities, such as prefrontal areas. Whilst the boundaries in such regions may not be congruent with architectonic domains, they were still useful in demonstrating that regional variance can be observed across the cortex using dMRI. Further analysis, involving high-resolution architectonic mapping is needed to shed light on what is driving the contrast in these areas. Such studies may also provide better training labels that can minimise the circular reasoning associated with supervised classification.

It should also be considered that we used only one of the many competing atlases. We remain some distance from being able to generate a definitive *in vivo*, cyto- and myeloarchitectonic reference map of the entire cortical surface. However, the method adequately demonstrated that diffusion MRI represents a complementary modality for future studies of cortical microstructure.

### Feature set comparison

The classification efficacy of several diffusion-based feature sets were assessed. In particular, we wanted to determine whether either (a) the explicit inclusion of radial and tangential diffusion properties (via the local surface normal) or (b) the use of higher-order feature sets, improves between-area contrast compared to commonly used scalar metrics, such as FA.

#### Binary S1 vs M1 classification

Initial tests on S1/M1 classification found relatively poor performance in lower order feature sets that only used a single b-shell or combined b-shells before classification (DT3, ND3). The consistently poor performance of DT3 compared to DT6 and DT9 supports the notion that different b-values can probe different aspects of cortical microstructure (Nagy et al., 2014). The relatively weak performance of ND3 suggests that the three-shell data contains more useful information than is captured by the NODDI model, which imposes biophysical assumptions regarding the underlying tissue composition. Crucially, the improvement in classification when adopting the second-order diffusion tensor (DT9), compared to the higher-order 4-tensor (4T36) or spherical harmonics (SH27), relies on the inclusion of surface specific metric, i.e., the radiality index, which was omitted from DT6.

**Fig. 9:**
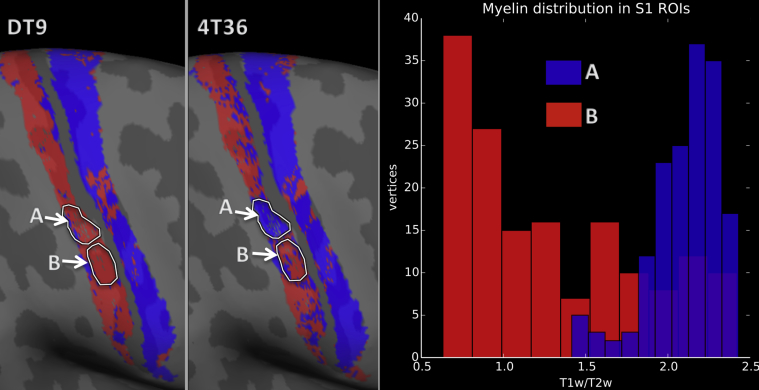
Confirmation that misclassification by the 4T36 feature set is driven by heterogeneity in myelin density within area 3b. The binary classification result for DT9 (left) and 4T36 (center) is shown with the outlines of the two ROIs that were selected. The myelin distribution within each ROI is also displayed (right), where myelin density is measured from the T1w/T2w ratio of the same subject.

The DT-based feature sets perform well despite diffusion at higher b-values (b > 1000) not respecting the Gaussian assumptions of this model (Alexander et al., 2002, Le Bihan et al., 2006). One possible interpretation is that the higher-order features may be driven more by noise and inter-subject variation than intrinsic features of distinct grey-matter regions, at least at our current resolution. Alternatively, we hypothesised that the low-order features are so distinct between these regions that they dominate the classification. The DT-based features also create a smoothing effect that results from the coarser description of the microstructure that they provide. As such, it is possible that DT model is insensitive to real, within area, microstructural variation. For example, there are myelin density changes corresponding to the boundaries of individual digits in 3b (Sereno and Tootell, 2005), or the hand, foot, and face subdivisions of areas 3b and 4 (Kuehn et al., 2017). Fig. 9 indicates that areas of misclassification by 4T36 correspond to large variations in the underlying myelin density (and thus architecture) within the tissue. These variations are not reflected in the DT results.

#### Full hemisphere parcellation

The full hemisphere, group average, and single subject results provide evidence in favour of using the more generalized, higher-order spherical harmonic feature sets. In contrast to the trend observed in the binary experiment, we found that in this multi-class problem SH27, not DT9, obtains the most accurate definition of 3b. It is possible that the DT-based features are not sensitive enough to describe differences between the larger set of classes. S1 has 3 neighbours in this 4-way classification meaning the three times as many features that SH27 provides are beneficial.

The more generalized features of SH27 provide a more coherent clustering result across the cortex, particularly for the single subject result. This again suggests that these features describe cortical tissue domains more effectively. Perhaps the higher order feature sets are less influenced by confounding effects, such as changes in global diffusivity between subjects, than the DT feature set as a result of their increased dimensionality. Support for the higher order feature sets is again evidenced in the distribution of areas in which SH27 out performs DT6 and DT9. It achieves better results for areas of high myelination, primary areas, or areas for which the training labels can be considered more reliable. On the other hand, it is possible that the SH27 feature set is more susceptible to overfitting in regions where the diffusion signatures of neighbouring areas are less distinct from each other. The combination of DT9 and SH27 can also be used as a feature set to maximise performance across the cortex (see Fig. A2). A few regions are still better classified with either of the reduced sets (DT9 or SH27) than with the combined feature set (SH27 + DT9). For example, area 3b is better classified using SH27 alone, suggesting that combining it with DT9, which performed comparatively poorly in this area (see Fig. 3B), adds noise to the RFC.

#### Inclusion of surface specific features

The inclusion of features that take the orientation of the local cortical surface into account (i.e., the radiality index in DT9, several features in the SH27 set), consistently offered an advantage over the scalar DT6. This is clear when comparing DT6 to DT9 in both the binary classification and group average experiments. However, we cannot conclusively say that such features are always necessary. Comparing the results of SH27 to 4T36 indicates that explicit reference to the local tissue orientation might not be required if the feature set provides a functionally complete description of the ADC. Further testing of 4T36 across a broader set of areas is required to confirm this.

Ultimately, deciding which feature set to use requires a nuanced approach that considers the specific aims of future studies. If attempting to delineate architectonic domains, the above results indicate that high order decomposition approaches might be more appropriate when describing the texture of the 'fabric' of the neuropil at an intermediate scale. On the other hand, an advantage of biophysical models is that they provide features that are more readily interpreted. For example, they are more useful if one wishes to understand the specific microstructural changes at the level of single fibres that can affect abnormal brain function.

The above works omit a set of recently emerging techniques, which aim to separate the contribution of microstructural tissue composition from the mesoscopic orientation distribution within the dMRI signal (Kaden et al., 2016, Reisert et al., 2017). These methods remain to be tested in a cortical parcellation framework and may be particularly beneficial in extrastriate or other non-primary areas that do not exhibit distinct tangential or radial laminar properties.

##### Limitations and future work

One of the limiting factors of the above work was the relatively coarse resolution of the diffusion data compared to the thickness of cortical laminae. This only allowed data to be sampled at a single cortical depth, which may have led to a failure to sufficiently capture variations in laminar structure, particularly for thicker areas of the cortex i.e., gyral crowns or area 4 (M1). It was also insufficient for reliably characterising the properties of V1. The relatively large voxels may have introduced noise by differential mixing of signals from different laminae in different locations. A finer sampling of different depths in each cortical column has the potential to provide a closer approach to the classical histological analysis of the cortex. The low resolution is also likely to incur partial volume effects, which we aimed to minimise by sampling at the middle cortical depth. However, further analysis in needed to determine the extent to which partial voluming from WM and CSF impact the results. The higher spatial resolutions that can be obtained at 7T have great potential for resolving these issues and validating the above findings.

There are several avenues that could be explored in more depth in future studies. For example, the labels were back-projected from a reference brain, but it would be interesting to investigate whether generating labels on individual HCP subjects can improve accuracy. Furthermore, we found that the feature ranking information provided by the RFC was highly variable between different regions – additional analysis could shed light on which features are more discriminative for which areas.

The results so far have concentrated on one hemisphere, but there are many interesting questions that will be able to be addressed in future studies where homologous regions are compared across hemispheres, given the well-documented differences in function between them.

## Conclusions

Our results provide support for including surface-based HARDI data analysis as an additional, independent measure of cortical microstructure to aid e.g. quantitative T1, which has been widely used as a proxy for myelin density. We demonstrated that higher-order decomposition methods provide a more consistent characterisation of grey matter microenvironments in regions for which the classification method can be considered most reliable. However, even simple lower order models such as the diffusion tensor provide contrast between cortical areas. In particular, combining the traditional diffusion tensor metrics of FA and MD with the surface specific radiality index is very powerful in binary classification between M1 and S1. Further work at higher resolutions and improved SNR will likely enhance the performance of these methods. With expected advances in data acquisition methods, it is likely that surface-based analysis of grey matter diffusion will become a new standard tool for probing microstructural variations among the complex mosaic of distinct cortical areas that make up the human neocortex.

## Figures and Tables

**Fig. A1:**
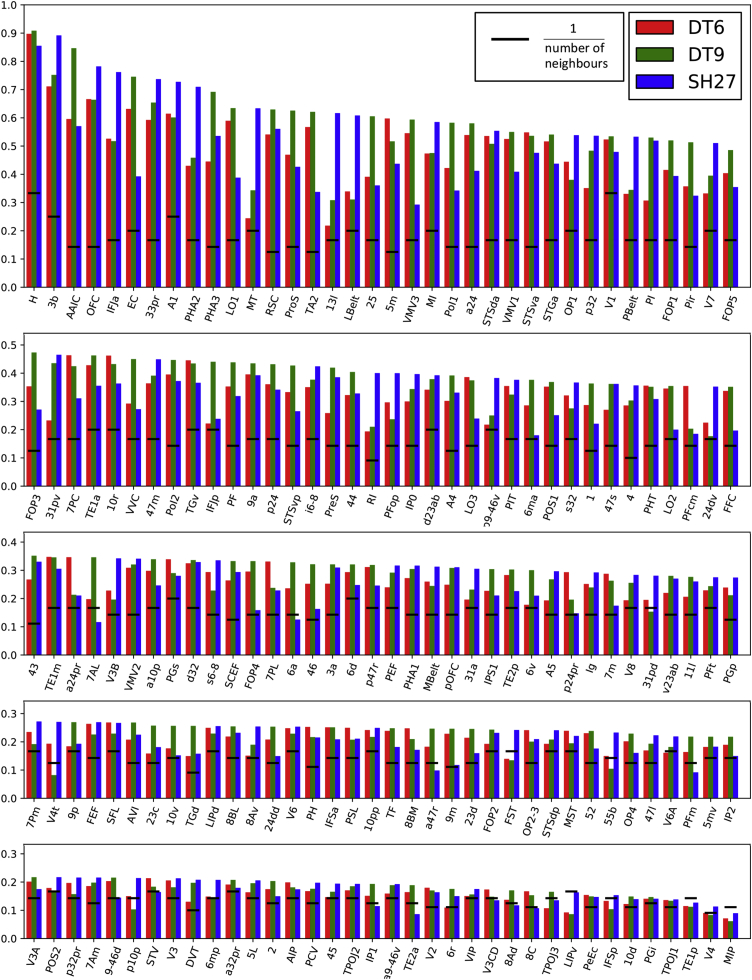
Bar charts comparing classification accuracy across all labels in the group average whole hemisphere parcellation. As in Fig. 5, the bars correspond to the proportion of correctly classified vertices in each ROI for feature sets DT6 (red), DT9 (green) and SH27 (blue). The ROIs have been ordered according to the overall highest classification accuracy across any of the feature sets. A comprehensive legend containing area names and descriptions can be found in the supplementary material of (Glasser et al., 2016).
